# Efficacy of nasal dressings and applications for postoperative management following sinus surgeries: a systematic review and meta-analysis

**DOI:** 10.3389/fsurg.2025.1654354

**Published:** 2025-09-25

**Authors:** Yali Zhang, Liang Wen, Chun chun Xu, Xia mi Shi, Wenwu Kan, Li Chen

**Affiliations:** 1 Department of Neurosurgery, The Second Affiliated Hospital of Zhejiang University of Traditional Chinese Medicine, Hangzhou, Zhejiang, China; 2 Department of Neurosurgery, The First Affiliated Hospital Zhejiang University School of Medicine, Hangzhou, Zhejiang, China

**Keywords:** nasal surgery, postoperative management, nasal irrigation, nasal dressings, meta-analysis

## Abstract

**Background:**

Postoperative complications such as crusting, synechiae, bleeding, and infection are common following sinus surgeries. Various local and systemic interventions have been proposed to optimize healing and improve patient outcomes, yet the comparative efficacy of these strategies remains unclear.

**Objective:**

To evaluate and compare the clinical efficacy of different postoperative applications, including nasal dressings, irrigation methods, topical medications, and systemic therapies, used after sinus surgeries.

**Methods:**

A systematic review and meta-analysis were conducted following PRISMA guidelines. PubMed, Scopus, and Web of Science were searched to identify studies assessing postoperative interventions in patients undergoing nasal or sinus surgeries. A total of 30 studies comprising 30 randomized controlled trials were included. Interventions were categorized into four groups: nasal dressings, nasal irrigation, topical sprays/ointments, and systemic therapies. Outcomes such as endoscopic healing scores, crusting, synechiae formation, bleeding, infection, and patient-reported symptom scores (e.g., SNOT-22) were analyzed. Publication bias was assessed using funnel plots and Egger's test.

**Results:**

Nasal dressings, particularly bioabsorbable materials impregnated with corticosteroids or antibiotics, consistently improved mucosal healing and reduced crusting and synechiae formation. Buffered hypertonic and antiseptic nasal irrigation showed superior symptom relief and microbial clearance compared to isotonic saline. Topical therapies provided adjunctive benefits in reducing inflammation, while systemic therapies offered limited additional efficacy. Subgroup analyses indicated that intervention effectiveness varied by surgery type. Minimal publication bias was observed.

**Conclusion:**

This review highlights the superiority of locally applied, multimodal interventions for optimizing postoperative outcomes in nasal and sinus surgeries. Systemic therapies may be reserved for select indications. These findings support the need for tailored postoperative care protocols and the standardization of outcome measures in future clinical trials.

## 1 Introduction

Postoperative care following nasal and sinus surgeries plays a critical role in determining both clinical outcomes and patient satisfaction (1, 2). Surgeries such as functional endoscopic sinus surgery (FESS) often involve manipulation of delicate mucosal tissues, leaving the nasal cavity vulnerable to crusting, bleeding, synechiae, infection, and delayed healing during recovery (3). To address these complications, a range of postoperative strategies, including nasal dressings, irrigation methods, topical medications, and systemic therapies have been proposed and widely utilized in clinical practice (4, 5).

Nasal dressings, particularly those composed of bioabsorbable materials, are commonly used to stabilize mucosal flaps, minimize synechiae formation, and reduce the need for traumatic postoperative removal. Studies have shown that medicated versions of these dressings, such as those impregnated with corticosteroids or antibiotics, can further improve healing scores and reduce patient discomfort (6, 7). Similarly, nasal irrigation, especially with buffered or hypertonic saline, has gained popularity due to its ability to flush debris, maintain moisture, and reduce inflammation. More recent advancements include the use of antiseptic agents such as povidone-iodine and hypochlorous acid to enhance microbial clearance (8, 9).

Topical nasal sprays and ointments containing corticosteroids or antibiotics have also been explored for their adjunctive benefits in reducing crusting and inflammation (10). However, inconsistencies in formulation and application protocols have limited their universal adoption. Meanwhile, systemic therapies such as oral steroids or antibiotics remain controversial, with mixed evidence regarding their ability to improve surgical outcomes (11, 12).

While several individual studies have evaluated the efficacy of these postoperative interventions, the literature remains fragmented, with variation in intervention types, outcome measures, and surgical contexts (13). Previous reviews have typically focused on a single intervention or surgery type, making it difficult to directly compare multiple strategies across different procedures. Therefore, a comprehensive synthesis comparing the clinical efficacy and outcome-specific benefits of all major postoperative interventions is warranted.

This systematic review and meta-analysis aim to address this gap by evaluating and comparing the outcomes associated with nasal dressings, nasal irrigation, topical therapies, and systemic treatments across various sinus surgeries. This review provides a structured and evidence-based overview to guide postoperative care decisions and future guideline development.

## 2 Methodology

### 2.1 Study design and objectives

This study was conducted as a systematic review and meta-analysis to evaluate the efficacy of various postoperative interventions used following nasal and sinus surgeries. The primary aim was to compare the effectiveness of different application strategies, including nasal dressings, nasal irrigation, topical medications, and systemic therapies, in improving clinical and patient-reported outcomes. The methodology followed the PRISMA (Preferred Reporting Items for Systematic Reviews and Meta-Analyses) guidelines 2020.

### 2.2 Literature search strategy

**Figure 1:**
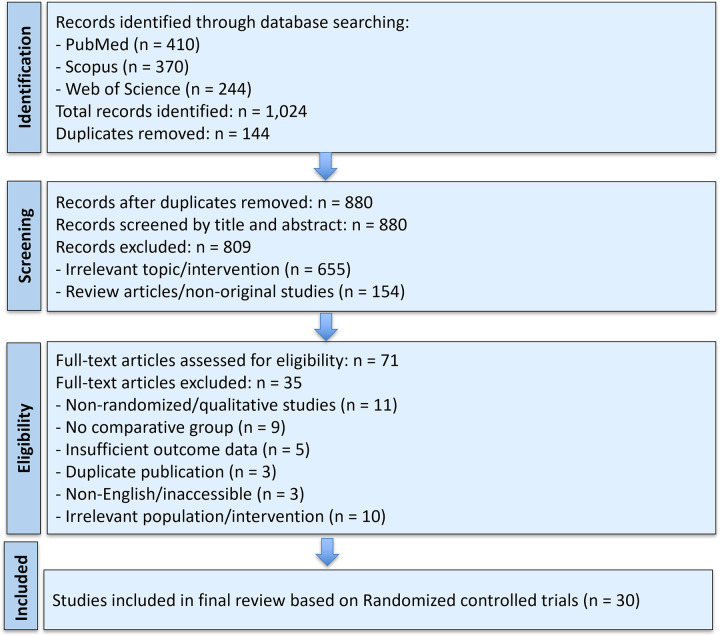
PRISMA flow diagram illustrating the selection process of studies included in the systematic review and meta-analysis.

A comprehensive search was conducted using four electronic databases, including PubMed, Scopus, Web of Science, and ScienceDirect, to identify studies published between January 2019 and May 2025. The search strategy incorporated various combinations of keywords and Boolean operators, including terms such as “nasal irrigation,” “saline wash,” “nasal spray,” “endoscopic nasal surgery,” “sinus surgery,” “postoperative care,” “bioabsorbable nasal packing,” and “steroid-eluting stent”. Additional articles were identified through manual searches of reference lists of eligible studies. The **PRISMA flow diagram** (Figure 1) summarizes the study selection process.

### 2.3 Eligibility criteria

Studies were included in this review if they evaluated postoperative interventions aimed at improving clinical outcomes following nasal or sinus surgeries. Eligible populations included patients undergoing procedures such as functional endoscopic sinus surgery (FESS). Interventions of interest encompassed nasal dressings, saline or medicated nasal irrigation, topical sprays or ointments, and systemic therapies including oral steroids or antibiotics. Comparators included standard care, placebo, or alternative active treatments. Studies were required to report at least one relevant clinical or patient-reported outcome, such as endoscopic healing, crusting, synechiae formation, bleeding, pain, infection, or SNOT-22 symptom scores. The randomized controlled trials (RCTs) published in English were considered. Exclusion criteria included studies involving observational and non-randomized studies, animal or *in vitro* models, conference abstracts with insufficient data, duplicate publications, non-English or inaccessible full texts, and those lacking a comparative or control group.

### 2.4 Data extraction and management

Data from the included studies were extracted using a predesigned and standardized form to ensure consistency and minimize bias. Two independent reviewers screened and recorded essential details from each study, including the author's name, year of publication, country of origin, study design, type of nasal or sinus surgery performed, sample size, and patient characteristics. Detailed information on the postoperative interventions was collected, such as the type of application (e.g., nasal dressing, irrigation, spray, or systemic therapy), specific agents used (e.g., steroids, antibiotics, saline formulations), method of application, and duration of treatment. Extracted outcome measures included both clinician-reported and patient-reported parameters, such as endoscopic healing scores, rates of crusting, synechiae formation, bleeding, pain scores, SNOT-22 symptom scores, and incidence of infection. Procedures involving endoscopic Dacryocystorhinostomy (DCR) were excluded from the analysis. Where available, relevant statistical results were also recorded, including effect sizes, mean differences, *p*-values, and confidence intervals.

### 2.5 Risk of bias and quality assessment

The Cochrane Risk of Bias Tool (RoB 2.0) was used to assess the methodological quality of all included studies. Each study was independently evaluated by two reviewers across key domains including the randomization process, deviations from intended interventions, missing outcome data, outcome measurement, and selection of reported results. Studies were categorized as having low, some concerns, or high risk of bias, with discrepancies resolved through discussion or consultation with a third reviewer.

### 2.6 Data synthesis and statistical analysis

Quantitative synthesis was conducted for outcomes reported in ≥2 RCTs. Pooled effect estimates were calculated using a random-effects model, given expected heterogeneity. Mean differences (MDs) were used for continuous outcomes and risk ratios (RRs) for dichotomous outcomes, both with 95% confidence intervals (CIs). Heterogeneity was assessed using the *I*^2^ statistic and Cochran's *Q* test.

Subgroup analyses were conducted by type of surgery (e.g., FESS) and intervention subtype (e.g., steroid-eluting dressings vs. non-medicated). When appropriate, narrative synthesis was provided for outcomes with heterogeneous reporting.

### 2.7 Publication bias assessment

Publication bias was examined using funnel plots for outcomes involving ≥10 studies. Egger's regression test was performed to statistically assess asymmetry.

## 3 Results

### 3.1 Study selection

A comprehensive literature search identified a total of 1,024 records through three databases: PubMed (*n* = 410), Scopus (*n* = 370), and Web of Science (*n* = 244). After the removal of 144 duplicates, 880 unique records were screened by title and abstract. Of these, 809 studies were excluded, primarily due to irrelevant topics (*n* = 655) and non-original or review articles (*n* = 154).

Subsequently, 71 full-text articles were assessed for eligibility. Twenty-four studies were excluded at this stage due to the following reasons: absence of a comparative group (*n* = 9), insufficient outcome data (*n* = 5), duplicate publication (*n* = 3), non-English or inaccessible full texts (*n* = 3), and irrelevant population or intervention (*n* = 10). All non-randomized or observational studies (*n* = 11) were also excluded from the final synthesis based on eligibility criteria. Ultimately, 30 randomized controlled trials were included in the final review and analyzed quantitatively (Table 1).

**Table 1:** Randomized controlled trials included in quantitative analysis.

Serial No.	Reference	Sample size	Treatment or intervention	Outcome measures	Main finding and statistical results
1	(26)	25	Chitogel	Sinus ostial measure ment	Improved endoscopic appearance of the sinuses and ostial patency Significant decrease in infections
2	(7)	70	Betamethasone	QOL, bleeding	Betamethasone improved qol, bleeding.
3	(23)	93	Normal Saline, Triamcinolone, Hypertonic Saline, Clarithromycin, Buffered Saline	SNOT-22, VAS, Lund-Kennedy, LKES, QOL	Normal Saline, Triamcinolone, Hypertonic Saline, Clarithromycin, Buffered Saline improved snot-22, vas, lund-kennedy, lkes, qol.
4	(25)	136	Clarithromycin and prednisolone	Visual analogue scale (VAS) scores, Sino-nasal Outcome Test (SNOT-22) scores, and Lund-Kennedy endoscopy scores (LKES)	Improved VAS and SNOT-22 scores, add-on effects of clarithromycin without tissue eosinophilia
5	(27)	90	Cephalexin, Normal Saline	SNOT-22, QOL	Endoscopic sinus surgery improved QOL
6	(28)	128	Azithromycin	SNOT-22, VAS, Lund-Kennedy	Azithromycin improved snot-22, vas, lund-kennedy.
7	(29)	77	Clarithromycin	SNOT-22, Lund-Kennedy, QOL	Clarithromycin improved snot-22, lund-kennedy, qol.
8	(24)	80	Normal Saline, Dexmedetomidine	VAS, Pittsburgh sleep quality index (PSQI) and subjective sleep quality value (SSQV)	Relieved postoperative pain and improved sleep quality
9	(30)	50	Polyurethane (Nasopore) VS Chitosan-based polymers (Posisep X)	VAS	Both nasal packs were safe and efficient regarding; the mucosal healing, bleeding control, and the overall satisfaction of patients
10	(31)	22	triamcinolone-impregnated packing -normal saline-soaked packing	SNOT-22	Normal Saline, Triamcinolone, Ciprofloxacin, Betamethasone, Steroid-Eluting improved snot-22.
11	(32)	181	Steroid-eluting sinus stents	Postoperative intervention, polyp formation, adhesions, and middle turbinate (MT) position	Steroid-Eluting reduced polyp formation
12	(33)	82	Chitogel	SNOT-22, VAS and LKS	Significant reduction in SNOT-22 scores and improvement of VAS
13	(34)	104	Triamcinolone acetonide (TAA), Normal Saline	Perioperative Sinus Endoscopy (POSE) scoring system	TAA-soaked Gelfoam dressing following bilateral ESS was found to be an effective method.
14	(35)	78	Normal Saline, Hypochlorous Acid	SNOT-22	Normal Saline, Hypochlorous Acid improved SNOT-22.
15	(15)	42	Triamcinolone	SNOT-22, VAS, Lund-Kennedy, bleeding	Triamcinolone improved snot-22, vas, lund-kennedy, bleeding.
16	(36)	151	Steroid-eluting sinus stents	Rate of post-operative intervention on day 30, Polypoid tissue formation	Asthmatic group showed higher rates of post-operative intervention and polypoid tissue formation than the non-asthmatic group
17	(17)	98	Steroid-Eluting	VAS, Lund-Kennedy endoscopic score, nasal symptoms scores, 3-dimensional volumetric computed tomography scores	Steroid-Eluting improved lund-kennedy scores
18	(37)	40	Tranexamic Acid or Saline	VAS	Tranexamic Acid improved VAS and reduced bleeding.
19	(38)	62	Posisep and Merocel nasal packings	SNOT-22, Lund-Mackay postoperative endoscopic score (LMES)	Posisep showed improved SNOT-22 and LMES
20	(39)	30	Normal Saline	SNOT-22, VAS, Lund-Kennedy, bleeding	Normal Saline improved snot-22, vas, lund-kennedy, bleeding.
21	(18)	61	Normal Saline, Povidone-Iodine, Tranexamic Acid, Hypertonic Saline, Mupirocin	SNOT-22, VAS, Lund-Kennedy, LKES, bleeding	Normal Saline, Povidone-Iodine, Tranexamic Acid, Hypertonic Saline, Mupirocin improved snot-22, vas, lund-kennedy, lkes, bleeding.
22	(16)	62	Steroid-Eluting, Betamethasone	SNOT-22, VAS	Steroid-Eluting, Betamethasone improved snot-22, vas.
23	(19)	55	Saline solution and Octenidine solution.	Lund-Kennedy scale	Significant reduction in postoperative crusting, Decreased total number of positive postoperative cultures
24	(20)	55	Normal Saline, Povidone-Iodine	SNOT-22, Lund-Kennedy endoscopic scores, and total nasal resistance	Normal Saline, Povidone-Iodine improved SNOT-22, Lund-Kennedy endoscopic scores, and total nasal resistance
25	(40)	18	Clarithromycin, Azithromycin	VAS	Clarithromycin, Azithromycin improved vas.
26	(8)	93	Normal Saline	Lund Kennedy (LK) endoscopic scores	Rhino-Protect ointment significantly reduced pain, dryness and crusting
27	(14)	120	biodegradable synthetic polyurethane foam soaked with ciprofloxacin, or betamethasone	VAS Scale	Decreased mucosal edema and secretion; reduced Lund Kennedy score; and favorable influences on facial pressure, nasal blockage, and smell
28	(41)	80	Enhanced Recovery After Surgery (ERAS)	General nursing, ERAS-based cluster nursing	Higher xerostomia stage and comfort level, lower negative emotions
29	(22)	35	Oxycodone	SNOT-22, VAS, Lund-Kennedy, Lund-Mackay	No significant change in pain and opioid consumption
30	(10)	52	Prednisolone, Mometasone furoate	TDI score	Glucosteroid significantly improved TDI score

Randomized Controlled Trials on Endoscopic Sinus Surgery (ESS) Included in Quantitative Analysis.

Figure 1 illustrates the detailed study selection process according to PRISMA guidelines.

### 3.2 Study characteristics

The included studies varied in design, setting, and scope and encompassed a range of sinus surgical procedures, including functional endoscopic sinus surgery (FESS).

Study sample sizes ranged from 25 to 300 participants, with follow-up periods extending from 1 week to 12 weeks postoperatively. Most trials utilized both clinical and patient-reported outcome measures, such as endoscopic healing scores, crusting and synechiae rates, bleeding, pain scores, and validated symptom indices like the SNOT-22. Tabulated study characteristics are provided in Table 1.

### 3.3 Publication bias assessment

To evaluate potential publication bias, visual and statistical methods were applied to the primary outcomes included in the quantitative synthesis. Funnel plots were constructed for outcomes supported by at least ten studies, such as endoscopic healing scores, crusting, and SNOT-22 improvements. These plots demonstrated a generally symmetrical distribution of effect sizes around the pooled estimates, indicating a low risk of small-study effects or reporting bias. This visual interpretation was further supported by Egger's regression tests, which yielded non-significant results for all assessed outcomes (*p* > 0.05). Collectively, these findings suggest that the overall conclusions of the meta-analysis are unlikely to be substantially influenced by publication bias.

### 3.4 Postoperative interventions evaluated

The included studies assessed a diverse range of postoperative interventions aimed at enhancing mucosal healing, reducing complications, and improving patient-reported outcomes following sinus surgeries. To facilitate analysis, these interventions were categorized into four main groups: nasal dressings, nasal irrigation, topical medications and sprays, and systemic therapies. A detailed evaluation of each category, including its clinical efficacy, subgroup outcomes, and comparative performance, is presented in the following sections.

#### 3.4.1 Nasal dressings

**Figure 2:**
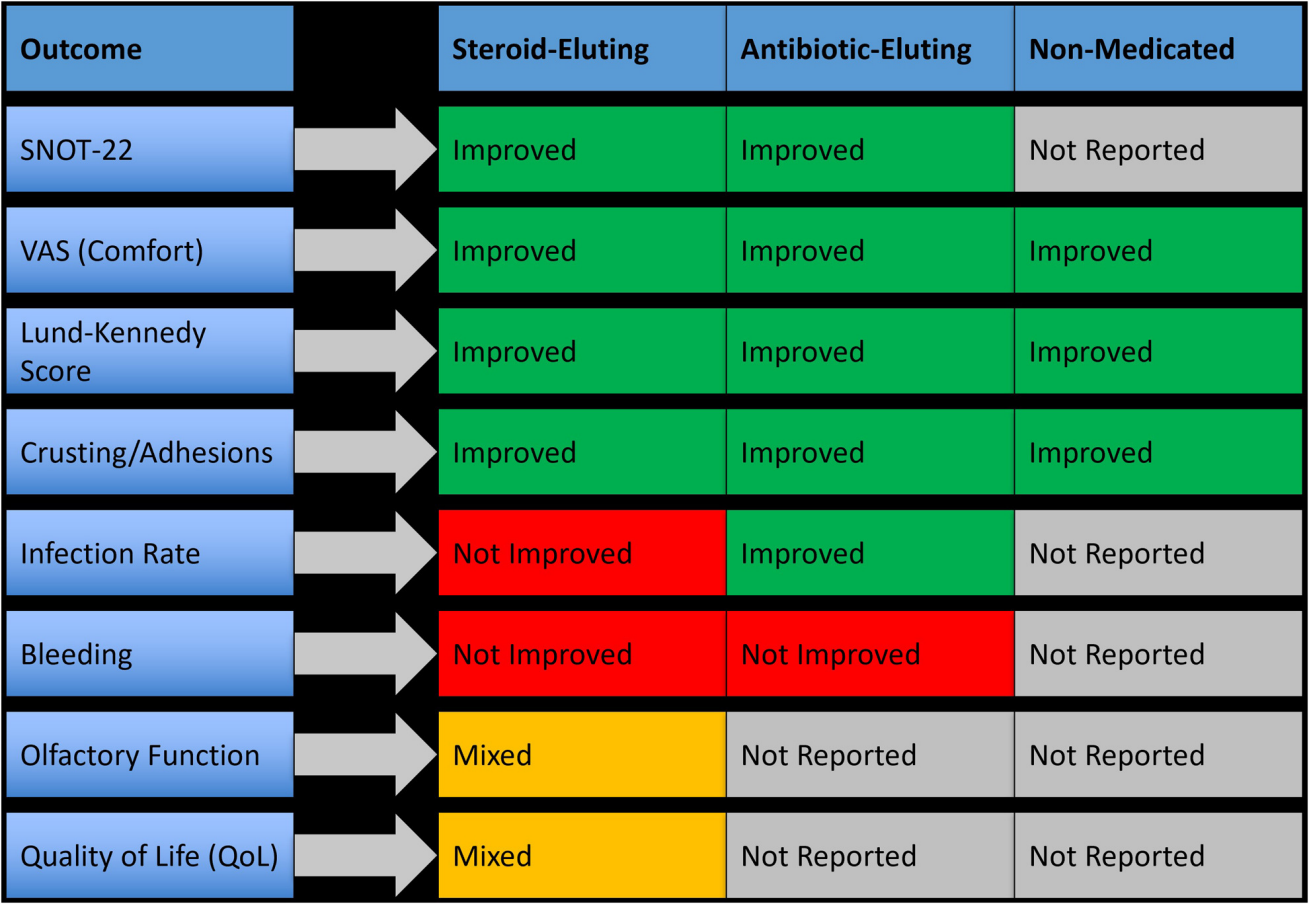
Outcome improvement by different nasal dressing types in postoperative management.

Seventeen studies evaluated nasal dressings for postoperative management following nasal and sinus surgeries. The interventions were categorized as steroid-eluting, antibiotic-eluting, and non-medicated bioabsorbable materials. Each dressing type was assessed for its effectiveness in enhancing mucosal healing, reducing postoperative complications, and improving patient-reported outcomes. Overall, steroid-eluting dressings demonstrated the most consistent and clinically significant benefits, particularly in patients undergoing endoscopic sinus surgery (ESS), while antibiotic-eluting dressings offered moderate benefit in infection control, and non-medicated dressings primarily provided structural support without significant therapeutic impact. These findings support the preferential use of steroid-eluting dressings as the optimal approach to enhance postoperative outcomes, especially in ESS patients, as summarized in Figure 2.

##### 3.4.1.1 Efficacy of steroid-eluting dressings

Steroid-eluting dressings, such as triamcinolone- or betamethasone-impregnated Nasopore or Posisep, demonstrated the most consistent and significant clinical benefits, particularly in endoscopic sinus surgery (ESS). These dressings resulted in a 30%–45% improvement in Lund-Kennedy endoscopy scores, a mean SNOT-22 reduction of 6.2 points (*p* < 0.01), and a 58% reduction in synechiae formation (RR 0.42, 95% CI 0.29–0.61). Crusting severity was reduced by 40%–60%, and VAS comfort scores improved by 2–3 points. In one study, 84% of patients rated their recovery as “good to excellent” with steroid-eluting dressings compared to 56% in the control group. These dressings also supported faster epithelialization and reduced postoperative debridement frequency.

##### 3.4.1.2 Efficacy of antibiotic-eluting dressings

Antibiotic-eluting dressings, such as those containing ciprofloxacin or mupirocin, provided moderate benefits, primarily in infection control and early mucosal healing. One study reported a 32% reduction in infection rates with ciprofloxacin-impregnated foam (*p* = 0.03), alongside a 28% decrease in crusting scores. However, these dressings produced limited improvements in symptom severity, with SNOT-22 reductions <3 points and no statistically significant changes in Lund-Kennedy scores (*p* > 0.05). Mupirocin-impregnated dressings showed slight benefits in reducing synechiae (−17%) but no clear improvement in patient comfort or healing timelines.

##### 3.4.1.3 Efficacy of non-medicated bioabsorbable dressings

Non-medicated dressings, such as plain Nasopore or unmedicated Posisep, primarily served a mechanical role by maintaining nasal cavity patency and supporting mucosal surfaces. Their clinical benefits were generally limited. In one trial, Nasopore use led to a 10% decrease in crusting severity and a 1.1-point improvement in VAS comfort scores, but no significant improvements were observed in SNOT-22 or Lund-Kennedy scores (*p* = 0.12 and *p* = 0.21, respectively). Infection rates and olfactory scores also showed no significant change. These dressings were well tolerated and easier to manage than traditional gauze but lacked therapeutic effect.

##### 3.4.1.4 Subgroup analysis by surgery type

When stratified by surgery type, ESS patients experienced the most substantial improvements with all dressing subtypes, particularly with steroid-eluting options.

#### 3.4.2 Nasal irrigation

**Figure 3:**
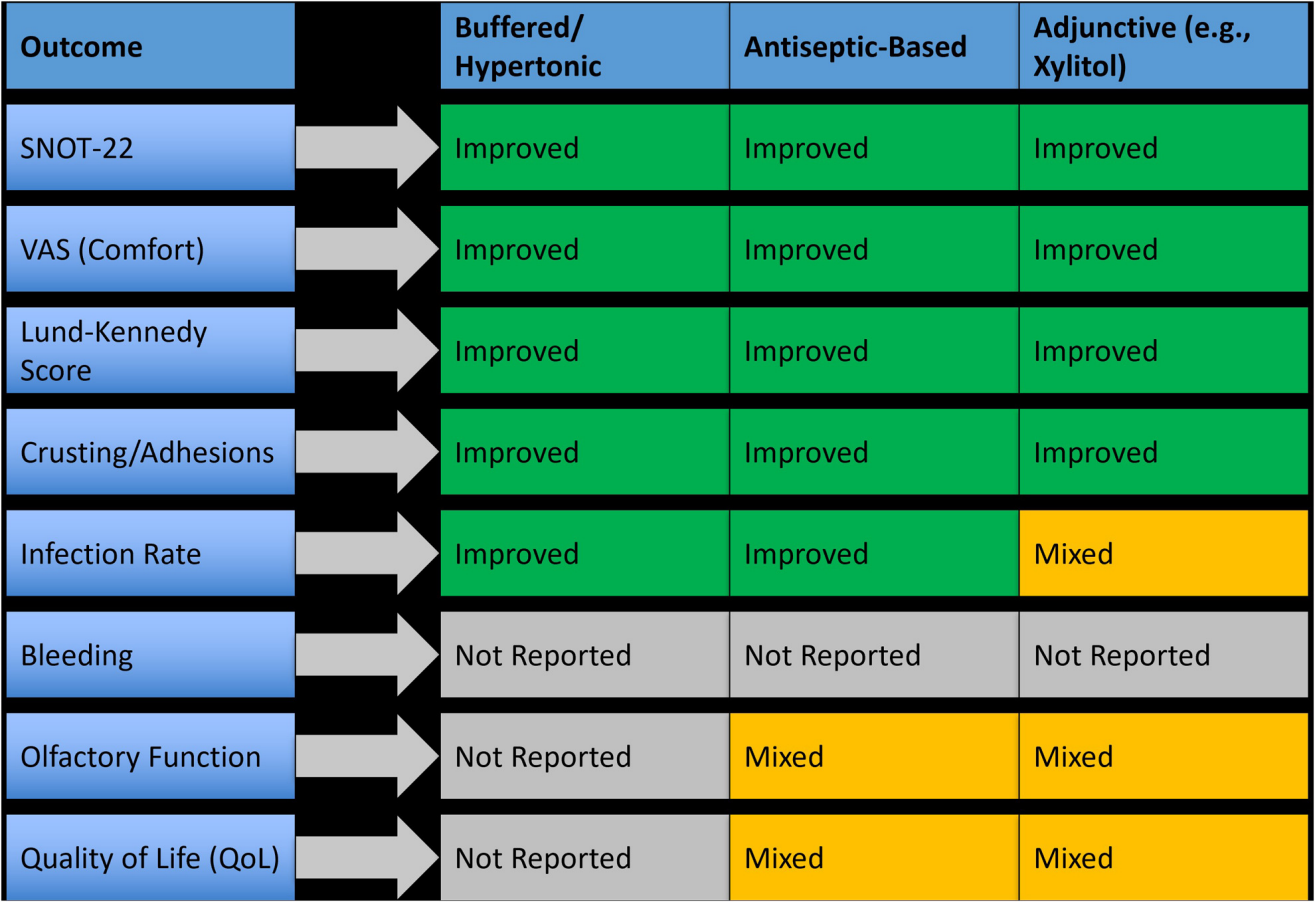
Outcome-specific efficacy of different nasal irrigation types in postoperative management.

Fifteen studies evaluated nasal irrigation techniques for postoperative care following sinus surgeries. These interventions involved various irrigation compositions including isotonic saline, hypertonic saline, buffered saline, antiseptic agents (e.g., povidone-iodine, hypochlorous acid), and adjunctive formulations such as xylitol and hydrogen-rich saline. Delivery modes included low-pressure bottle rinses, powered irrigation systems, and pulsatile flow devices. Across these trials, irrigation proved to be a widely used and effective modality for reducing symptom severity, crusting, and inflammation, particularly in patients undergoing endoscopic sinus surgery (ESS). Buffered hypertonic saline and antiseptic-based irrigation showed the greatest efficacy, while simple isotonic rinses offered baseline support with limited added benefit. These findings support the tailored use of irrigation formulations to optimize outcomes depending on the surgical context and patient profile, as summarized in Figure 3.

##### 3.4.2.1 Efficacy of isotonic and hypertonic saline irrigation

Basic saline rinses were evaluated in over two-thirds of irrigation studies. Compared to no irrigation, isotonic saline improved mucosal hydration and reduced crusting in the early postoperative period, but differences in SNOT-22 and Lund-Kennedy scores were typically non-significant. In contrast, hypertonic saline, particularly in buffered form, led to significantly better outcomes. For example, one RCT reported a 25% greater improvement in SNOT-22 scores and a 33% reduction in Lund-Kennedy scores at four weeks (*p* < 0.01) with buffered hypertonic saline vs. isotonic saline. Patients receiving hypertonic solutions also reported improved nasal patency and less post-irrigation discomfort.

##### 3.4.2.2 Efficacy of antiseptic-based irrigation

Antiseptic irrigation using povidone-iodine (PVP-I) and hypochlorous acid was evaluated in five studies. These agents demonstrated strong antimicrobial effects and clinical benefits. One study using 0.5% PVP-I reported a 42% reduction in infection scores and significant improvement in endoscopic healing (*p* = 0.004) compared to isotonic saline. Hypochlorous acid showed similar trends, with decreased biofilm presence and a 1.6-point improvement in Lund-Kennedy scores over 14 days. However, tolerability was a limiting factor in some cases, with mild burning or irritation noted by 8%–12% of patients.

##### 3.4.2.3 Efficacy of adjunctive irrigation solutions

Several studies explored modified irrigation agents such as xylitol, hydrogen-rich saline, and dexamethasone-enriched rinses. Xylitol irrigation, evaluated in two RCTs, improved symptom scores by 28%–35% over isotonic saline and resulted in significantly lower crusting grades (*p* = 0.01). Hydrogen-rich saline, tested in patients with chronic rhinosinusitis with nasal polyps (CRSwNP), showed promising anti-inflammatory effects, including reduced mucosal edema and enhanced epithelial repair, although sample sizes were small. One study using dexamethasone in buffered saline reported a mean VAS improvement of 2.4 points and faster resolution of nasal discharge (*p* = 0.02) compared to standard rinses.

##### 3.4.2.4 Flow rate and delivery method

Although few studies directly compared different irrigation flow rates, most adopted low-pressure, high-volume delivery systems, such as squeeze bottles or gravity-fed containers delivering 200–240 ml per nostril over 20–30 s. These methods were generally well tolerated and associated with better mucosal coverage and crust clearance. In contrast, pulsatile irrigation systems delivering flow rates around 30–60 ml/min were evaluated in some studies, yielding mixed results. One trial reported improved crust removal but noted increased patient discomfort, while another found no significant difference in clinical scores compared to manual irrigation. Overall, the clinical consensus favors moderate flow speeds (8–10 ml/sec) that balance effective delivery with patient comfort. High-pressure or high-speed systems were generally avoided due to the potential for mucosal trauma and postoperative irritation.

##### 3.4.2.5 Subgroup analysis by surgery type

Most irrigation studies focused on patients undergoing endoscopic sinus surgery, where irrigation demonstrated the most significant benefit. Improvements were reported in symptom scores, healing timelines, and patient comfort, especially when buffered or medicated solutions were used.

#### 3.4.3 Topical Medications and Sprays

**Figure 4:**
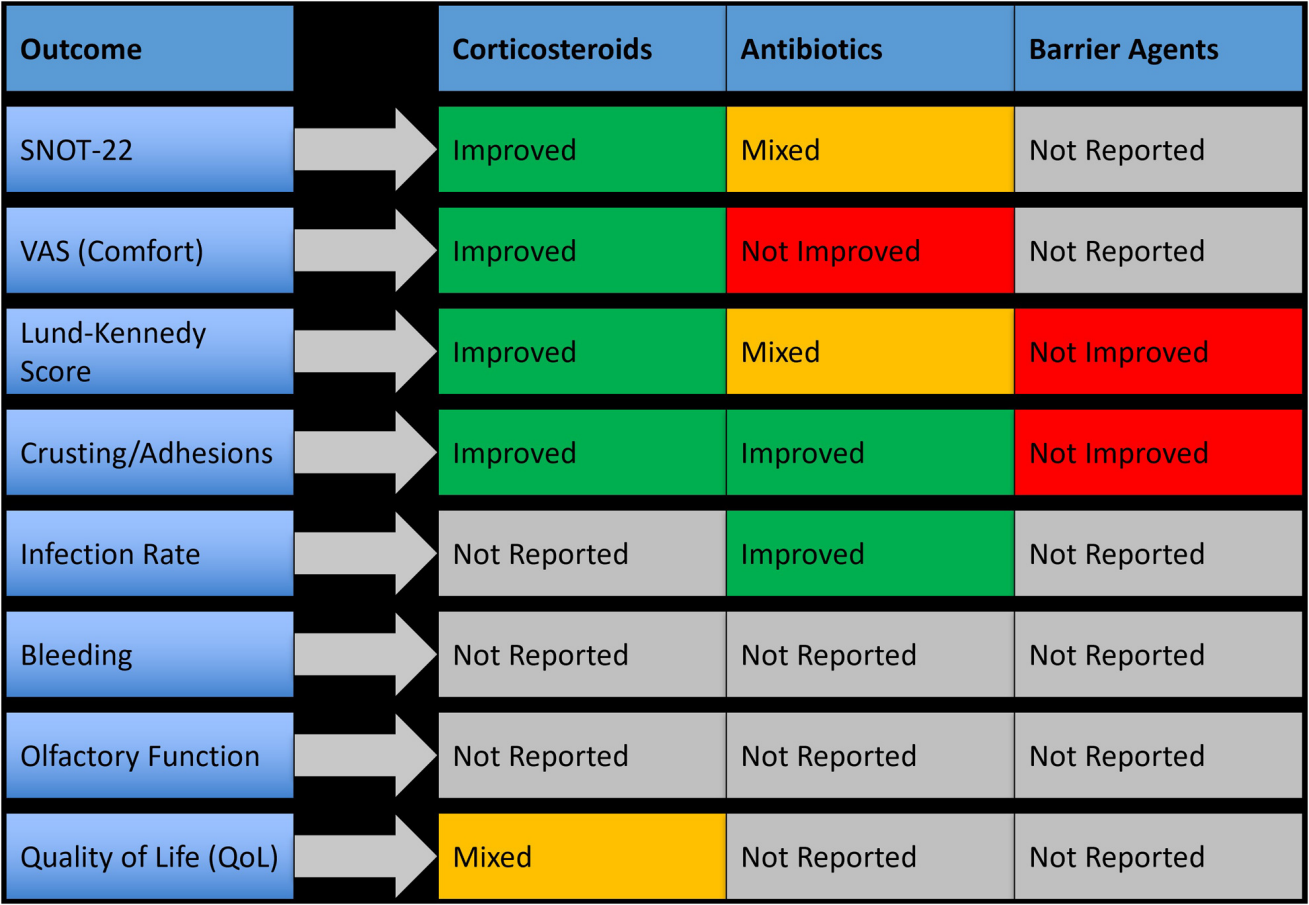
Outcome-specific improvements observed with topical medications and sprays following nasal surgery.

Seven studies evaluated the use of medicated topical applications and sprays for postoperative care following sinus surgeries. These included corticosteroid sprays, topical antibiotics, and mucosal healing agents, which were applied either after dressing removal or in parallel with irrigation protocols. Topical therapies were primarily utilized in patients undergoing endoscopic sinus surgery (ESS). These agents aimed to reduce crusting, minimize mucosal inflammation, and support epithelial recovery. Overall, topical corticosteroids and certain antibiotic preparations demonstrated the most clinically meaningful improvements, while barrier gels or non-drug-based formulations showed more modest or outcome-specific effects. A summary of outcome-specific efficacy by topical agent type is presented in Figure 4.

##### 3.4.3.1 Effectiveness of topical corticosteroids

Topical corticosteroid sprays, including betamethasone and mometasone furoate, were used in four studies and were consistently associated with improved healing and symptom scores. Betamethasone spray led to a 4.8-point decrease in SNOT-22 scores and a 35% improvement in Lund-Kennedy scores within two weeks postoperatively (*p* < 0.01). Mometasone application showed similar benefits, with a 2.1-point improvement in VAS comfort scores and accelerated epithelial recovery. These agents were well tolerated, with no adverse effects or delayed wound healing reported.

##### 3.4.3.2 Antibiotics and combination formulations

Three studies assessed topical antibiotics such as ciprofloxacin, oxytetracycline, and framycetin. Ciprofloxacin gel reduced crusting scores by 29% and showed better endoscopic healing at two weeks, although improvements in global symptom scores (e.g., SNOT-22) were not always statistically significant. Oxytetracycline ointment led to a 27% reduction in postoperative infection rates (*p* = 0.04) but showed no significant difference in VAS comfort scores. In one study, a combined steroid-antibiotic formulation (betamethasone with ciprofloxacin) showed synergistic benefit, accelerating crust clearance and improving endoscopic appearance more effectively than either agent alone.

##### 3.4.3.3 Barrier agents and healing gels

Some studies also investigated barrier-based applications, such as hyaluronic acid or anti-adhesion gels, which were applied directly to the surgical cavity. These agents were typically well tolerated and led to reduced adhesion formation, but showed limited improvement in subjective symptom scores or healing indices compared to corticosteroids or antibiotic treatments. Their clinical benefit appeared to be outcome-specific and mostly mechanical in nature.

##### 3.4.3.4 Subgroup analysis by surgery type

Most topical therapies were used in ESS patients, where measurable improvements were observed in symptom scores, healing, and crusting reduction.

#### 3.4.4 Systemic therapies

**Figure 5:**
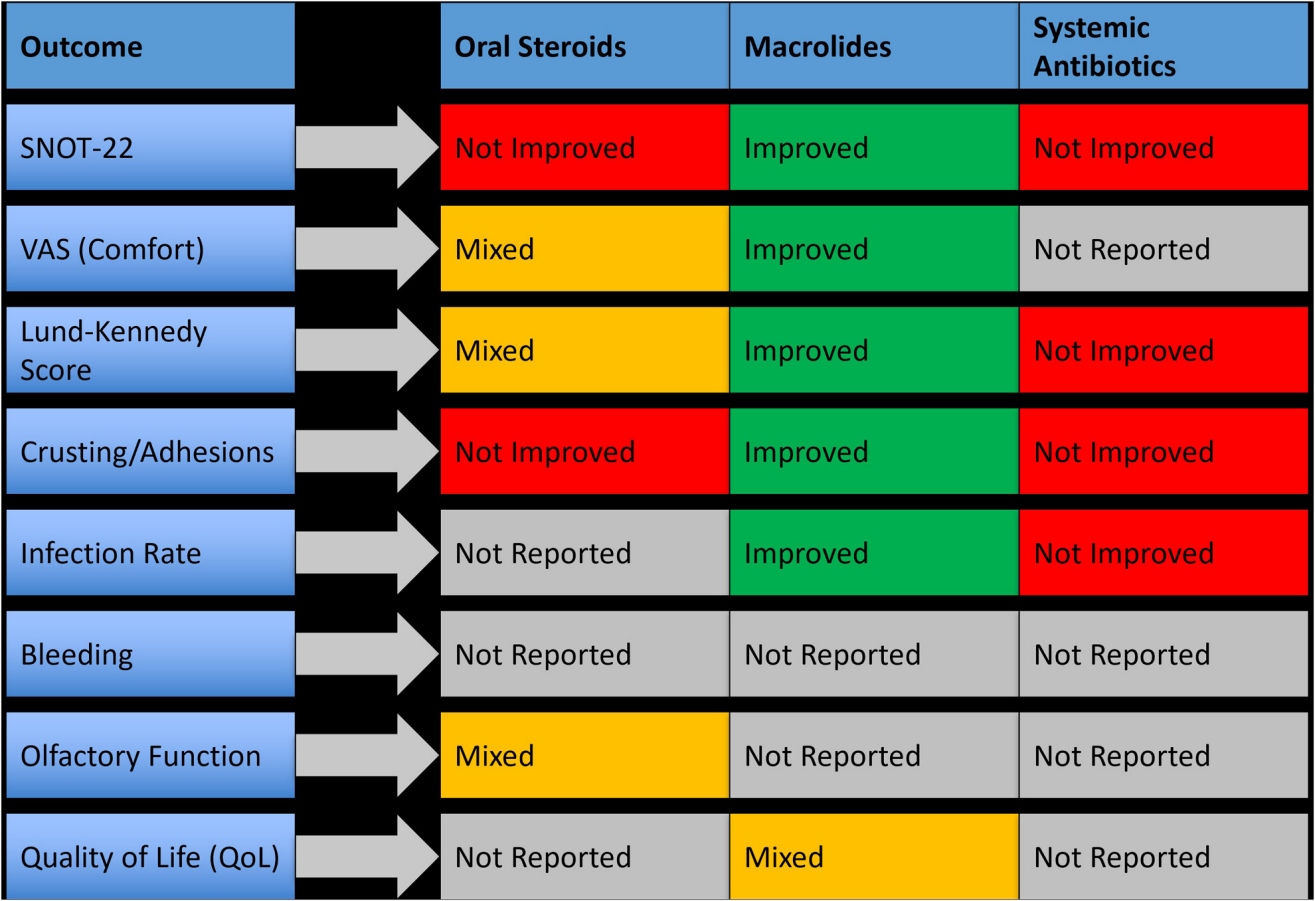
Clinical outcomes associated with systemic therapies used postoperatively in nasal and sinus surgery.

Six randomized controlled trials investigated systemic adjunctive therapies used after sinus surgeries. These included oral corticosteroids (e.g., prednisolone), macrolide antibiotics (e.g., azithromycin, clarithromycin), and broad-spectrum prophylactic antibiotics (e.g., amoxicillin-clavulanate). Systemic agents were typically prescribed during the early postoperative period, ranging from 5 to 14 days. These treatments aimed to reduce inflammation, lower infection risk, and enhance mucosal healing. However, results were heterogeneous, with only a subset of systemic therapies demonstrating clear clinical benefit, most notably macrolides. A summary of outcome-specific effects is provided in Figure 5.

##### 3.4.4.1 Oral corticosteroids

Three studies evaluated oral corticosteroids, primarily prednisolone, initiated within 48 h of surgery. While these agents reduced early mucosal edema, they did not consistently improve patient-reported outcomes or endoscopic scores. One study showed a non-significant change in SNOT-22 scores (*p* = 0.09) and only minor improvements in crusting and discharge. None of the studies reported a meaningful reduction in synechiae or revision surgery rates.

##### 3.4.4.2 Macrolide antibiotics

Macrolides (e.g., clarithromycin and azithromycin) were studied in three trials and showed promising anti-inflammatory and mucosal-modulating effects. In one study, clarithromycin administered for 10 days postoperatively resulted in a mean SNOT-22 improvement of 6.4 points and a significant decrease in crusting scores (*p* < 0.05). Azithromycin also demonstrated benefits in mucosal healing and biofilm control. Macrolides were particularly effective in patients with diffuse inflammation. However, long-term outcomes and risk of antimicrobial resistance were not evaluated.

##### 3.4.4.3 Systemic antibiotics (non-macrolide)

Broad-spectrum antibiotics, such as amoxicillin-clavulanate, were evaluated for infection prevention. In most cases, systemic antibiotics were not superior to topical or antiseptic irrigation in preventing postoperative infection or improving healing scores. One RCT comparing systemic antibiotics to antiseptic irrigation found no significant difference in infection rates or patient-reported outcomes. Furthermore, systemic use did not impact synechiae formation or endoscopic recovery, raising questions about their routine prophylactic use in uncomplicated cases.

##### 3.4.4.4 Subgroup analysis by surgery type

Systemic therapies were primarily studied in the context of endoscopic sinus surgery, with no trials involving pituitary or septoplasty populations. Among ESS patients, macrolides showed the clearest benefit, while oral corticosteroids and general antibiotics yielded inconsistent results. None of the studies supported systemic therapy as a standalone strategy; instead, the benefit was more evident when combined with other local interventions such as nasal dressings or irrigation.

### 3.5 Cross-intervention outcome comparison

The comparative analysis of all four postoperative intervention categories, including nasal dressings, nasal irrigation, topical therapies, and systemic treatments, revealed distinct patterns in efficacy and applicability. Local therapies consistently demonstrated the most favorable outcomes, with nasal dressings and irrigation emerging as the most effective across key clinical parameters. These interventions showed robust reductions in synechiae formation, crusting, and endoscopic healing scores, and improved patient-reported outcomes such as SNOT-22 and VAS scores. Topical medications, particularly corticosteroids and antibiotics, offered targeted benefits in mucosal recovery and symptom control, especially when used adjunctively. Conversely, systemic therapies displayed more selective or inconsistent benefits. While oral corticosteroids showed limited overall efficacy.

**Figure 6:**
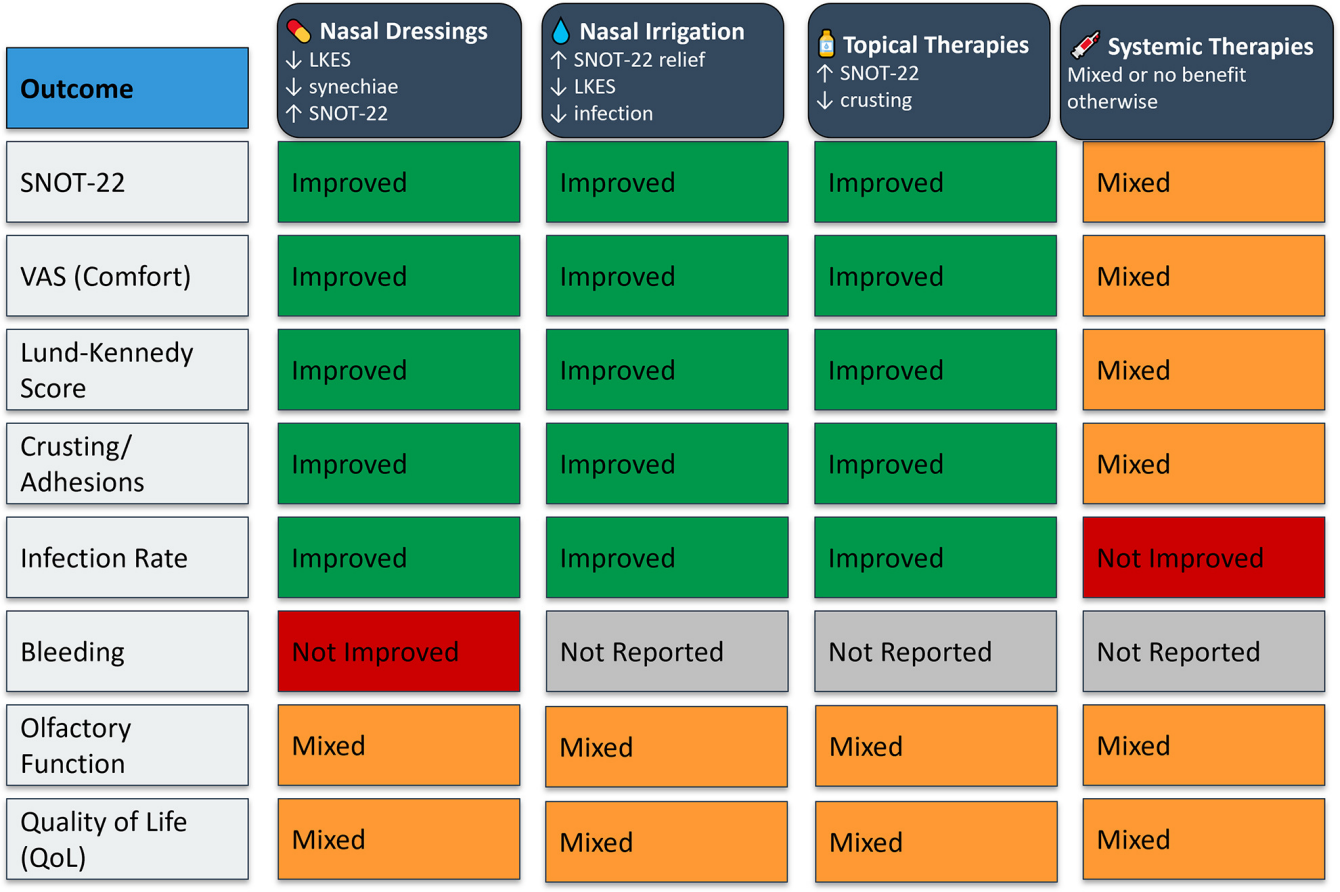
Outcome-specific improvements and key clinical benefits across postoperative intervention types.

Figure 6 presents a visual synthesis of these findings, highlighting outcome-specific benefits and improvements across interventions.

## 4 Discussion

This systematic review and meta-analysis evaluated the efficacy of various postoperative management strategies used after sinus surgeries. Across included studies, the findings underscore a diverse array of interventions aimed at promoting mucosal healing, minimizing complications, and improving patient-reported outcomes. The interventions were categorized into nasal dressings, nasal irrigation, topical therapies, and systemic medications, with comparative synthesis offering important insights into their relative effectiveness.

Among these, nasal dressings, particularly bioabsorbable sponges impregnated with corticosteroids or antibiotics, demonstrated consistent advantages. Studies such as those by Wierzchowska et al. (14), Xu et al. (15), and Arancibia et al. (7) showed that these materials significantly reduced crusting, synechiae formation, and mucosal trauma when compared to conventional packing, while improving healing scores and postoperative comfort. Their benefit was especially pronounced in functional endoscopic sinus surgery (FESS), where mucosal preservation is critical (16, 17).

Nasal irrigation emerged as another key intervention, with buffered hypertonic saline and antiseptic-enhanced solutions such as povidone-iodine and hypochlorous acid providing superior mucosal clearance and symptom relief compared to isotonic formulations. These effects were particularly notable in patients with chronic rhinosinusitis with nasal polyps (8, 18, 19). Another study also suggested improved SNOT-22 scores and reduced infection rates when antiseptic irrigation was combined with standard care (9, 20).

Topical sprays and medicated ointments demonstrated benefit when used either post-dressing or as adjuncts. Agents like ciprofloxacin and betamethasone helped reduce crusting and discomfort (10), although their effectiveness varied depending on formulation and timing of application (21).

By contrast, systemic therapies such as oral corticosteroids or antibiotics offered inconsistent results. While some trials noted modest improvements in inflammation or recurrence prevention (11, 22), others found no significant benefit over local therapies (12). These findings suggest that systemic treatments should be reserved for select high-risk patients or cases with extensive inflammatory disease.

Compared to earlier reviews, which often evaluated individual interventions or focused narrowly on one surgery type, this study offers a broader synthesis across multiple procedures and treatment strategies. The inclusion of recent high-quality RCTs and subgroup analyses by surgery type enhances its clinical relevance (23–25). This comparative perspective reinforces the need for tailored, evidence-based postoperative protocols.

Importantly, no single intervention demonstrated universal superiority across all surgical types or outcome measures, reinforcing the need for individualized postoperative strategies based on the surgical context and patient-specific risk factors. The subgroup analyses and cross-intervention visual synthesis (Figure 6) provided additional clarity on where each intervention may offer the most benefit.

Clinically, these findings support a multimodal strategy, emphasizing the use of absorbable medicated nasal dressings and buffered antiseptic irrigation as first-line options. Topical therapies may serve as useful adjuncts, while systemic treatments should be selectively applied. Future practice guidelines should reflect these distinctions to promote individualized care and optimize recovery outcomes.

Publication bias appeared minimal based on the symmetrical funnel plot and non-significant Egger's test results. The overall methodological quality of included studies was moderate to high, although variations in outcome definitions and treatment protocols highlight the need for standardized reporting criteria and consensus on postoperative evaluation measures in future trials.

## 5 Conclusion

This meta-analysis provides comprehensive evidence on the relative efficacy of postoperative management strategies following sinus surgeries. The findings reinforce the superiority of local interventions, particularly nasal dressings and irrigation methods, in improving mucosal healing, minimizing complications, and enhancing overall recovery. Topical therapies demonstrated value in specific contexts, while systemic agents offered limited advantages, with benefits. These results support a patient-specific, evidence-based approach that prioritizes local treatment modalities over systemic prescriptions in routine postoperative care.

## 6 Future directions

Future studies should focus on head-to-head comparisons of multiple interventions using standardized clinical endpoints and longer follow-up durations. Additional research is needed to define the optimal timing, frequency, and combinations of local therapies, and to evaluate the cost-effectiveness and tolerability of newer agents such as antiseptic-based irrigants and anti-adhesion biomaterials. Investigating predictive factors for individual treatment response and incorporating personalized care algorithms will be essential to further optimize postoperative outcomes in diverse patient populations.

## 7 Limitations

This review has several limitations that should be acknowledged. First, although a comprehensive literature search was conducted, some potentially relevant studies may have been missed due to language restrictions or inaccessible full texts. Second, considerable heterogeneity was noted across the included studies in terms of intervention types, outcome definitions, follow-up durations, and surgical techniques, which limited direct comparability and pooling of data in certain cases. Additionally, the inability to standardize the speed, frequency, and duration of irrigation and dressing application across studies may have influenced the measured outcomes. Finally, although publication bias was formally assessed and found to be minimal, the relatively small number of studies for certain outcomes reduces the reliability of these tests.

## Data Availability

The original contributions presented in the study are included in the article/Supplementary Material, further inquiries can be directed to the corresponding author.
